# Phase II study of docetaxel, cisplatin, and fluorouracil in patients with distantly metastatic penile cancer as first-line chemotherapy

**DOI:** 10.18632/oncotarget.4802

**Published:** 2015-07-30

**Authors:** Sheng Zhang, Yao Zhu, Dingwei Ye

**Affiliations:** ^1^ Medical Oncology, Fudan University Shanghai Cancer Center, Shanghai 200032, China; ^2^ Department of Oncology, Shanghai Medical College, Fudan University, Shanghai 200032, China; ^3^ Department of Urology, Fudan University Shanghai Cancer Center, Shanghai 200032, China

**Keywords:** penile cancer, docetaxel, fluorouracil, metastases

## Abstract

**Purpose:**

Patients with distantly metastatic (M1) penile squamous carcinoma have extremely poor prognosis and few prospective clinical trials evaluating systemic treatment have ever been performed for this population.

**Methods:**

Patients (aged ≥ 18 years) with histologically confirmed, distantly metastatic, measurable penile squamous carcinoma were enrolled. They were treated with docetaxel 75 mg/m^2^ (day1), cisplatin 70 mg/m^2^ (day1), and fluorouracil 500 mg/m^2^/d (days 1 to 5) every 3 weeks as first line chemotherapy. The primary endpoint was objective response rate (ORR).

**Results:**

39 patients received chemotherapy with a median of four cycles (range two to six). The median follow-up time was 11 months. 15 patients had a confirmed objective response (38.5%, 95% CI 23.36–55.38), all of which were partial responses. The median progression-free survival (PFS) was 3 months (95% CI 2.92–3.09), and the median overall survival (OS) was 7 months (95% CI 5.99–8.03). Toxicity was manageable and the most frequently recorded adverse events of grade 3 or higher were neutropenia (13 of 39; 33%), nausea/vomiting (7 of 39;18%). There was no treatment-related death.

**Conclusion:**

The palliative regimen of docetaxel, fluorouracil, and cisplatin induced moderate responses and can be used as a choice for the treatment of patients with distantly metastatic penile cancer. However, efforts to improve efficacy and minimize toxicity for this regimen should be made in the future.

## INTRODUCTION

Penile squamous cell carcinoma is a rare cancer in developed countries. In 2013, 1,570 new cases and 310 deaths from penile cancers were predicted to occur in the United States [1]. However, it is a serious health problem in developing countries. It can constitute up to 10% of malignant diseases in men in some African and South American countries, and this rate is 6% in some parts of India. In fact, penile cancer is the most commonly diagnosed cancer in some developing countries such as Uganda [2]. Worldwide, an estimated more than 26,000 new cases are diagnosed annually [1].

In addition to difference in overall incidence between developed and developing countries, the stage of penile cancer might be also substantially different. More patients with penile cancer at early stage in developed world were diagnosed and treated compared with those in developing countries [2–4]. It has been reported that the higher incidence and later stage of penile cancer in developing countries were possibly related to poor penile hygiene, more smoking habit, absence of circumcision, suboptimal availability of medical care, and other socioeconomic factors [5–7].

The rarity of penile cancer in developed countries made the conduct of prospective clinical trials on which to build clinical treatment was scarce. Particularly, the already limited trials for late stage penile cancer mainly recruited heterogeneous populations of patients with different prognoses in order to accrue sufficient number of patients [3, 8]. For example, in AJCC staging system, stage IV penile cancer comprises T4, or N3, or M1 patients. The incidence of distant metastases (M1) has been 1%–10% at the initial presentation or disease recurrence [4, 9, 10]. For the locally advanced T4 or N3 patients, it has been demonstrated that a significant proportion of patients could enjoy long disease free survival if they received neoadjuvant therapy and subsequent curative surgery. Overall survival (OS) for this population could be longer than two years [11–14]. However, for the M1 patients with distant metastases, palliative chemotherapy is the only effective treatment modality, and the prognosis for this population is extremely poor. It was reported in literature that the penile cancer patients at M1 stage only have overall survival of several months [4, 9]. Clearly the locally advanced T4 or N3 patients and distantly metastatic M1 patients require different treatment evaluations. However, previous studies usually combined T4, N3 and M1 patients with penile cancer for accrual purpose. This demonstrates an unmet medical need for this group of patients with extremely poor prognosis.

Recently, the combination of docetaxel, cisplatin and fluorouracil has shown high activity in head and neck squamous cancer and other cancer treatments which showed histopathologic similarity to penile squamous cancer [15, 16]. It was also shown that this combination chemotherapy was active in a few late stage penile patients in retrospective analysis [17]. In this study, we evaluated the efficacy and safety of a similar regimen in the treatment of M1 stage penile cancer in terms of conventional response rate, progression-free survival (PFS), OS, and toxicity.

## RESULTS

### Patients

Between November 2009 and July 2013, 39 patients with distantly metastatic penile cancer (M1 stage) were enrolled in our center in the study. All patients were assessed for efficacy and safety. The patients characteristics were listed in Table 1. The median patient age was 59 years (range from 34 to 75 years), and the distant metastases were mostly in the lung (30.8%), lymph nodes (38.5%), and the liver (25.6%). 61.5% patients had ECOG performance status 1.28% patients were included in this study to receive primary treatment for penile cancer. 64.2% patients received surgeries and recurred at the time of enrollment. Because the role of adjuvant chemotherapy after surgeries of penile cancer was not established, adjuvant chemotherapy was not recommended for these patients in our center. The patients enrolled in this study were chemotherapy naive.

**Table 1:** Patient demographics and clinical characteristics

Demographic or Clinical Characteristic	No. of Patients (*N* = 39)	%
Age, years		
Median	59	
Range	34–75	
ECOG performance status		
0	15	38.5
1	24	61.5
Skin ulceration	14	35.9
T stage		
Tx	11	28
T1	4	10
T2	9	23
T3	11	28
T4	4	10
N stage		
N1	9	23
N2	17	43.6
N3	11	28
Nx	2	5.1
Distant metastasis (M1)		
Lung	12	30.8
Liver	10	25.6
Pleural	5	12.8
Lymph nodes	15	38.5
Other soft tissue	7	18
Prior treatment		
Surgery	25	64.2
Radiation therapy	5	12.8
Presentation of disease		
Primary	11	28
Recurrent	28	72

Abbreviation: ECOG, Eastern Cooperative Oncology Group.

### Efficacy

Overall mean relative dose intensity was 91%. Median number of chemotherapy was 4 cycles (range: 2–6). The most frequent reasons for discontinuation were progressive disease (44%), adverse events (18%) and patient's decision not to receive further treatment (18%). Dose reductions occurred in 21% patients. Gastrointestinal toxicities were the most common adverse events leading to cycle delay.

The median follow-up time was 11 months. Of the 39 evaluable patients, no patients had a CR, and 15 patients had a confirmed PR, resulting in an overall response of 38.5% (95% CI 23.36–55.38). Another 14 patients showed stable disease as best response (35.9%). Thus disease control (CR plus PR plus SD) was noted in 30 (74.4%) patients (Table 2).

**Table 2:** Response to regimen (*N* = 39)

	No. of patients	%
Confirmed response		
Complete response	0	0
Partial response	15	38.5
Confirmed stable disease	14	35.9
Progressive disease	10	25.6
Overall response rate	15	38.5
95% CI for ORR		23.4–55.4

Abbreviations: CI, confidence interval. ORR, objective response rate.

**Figure 1:**
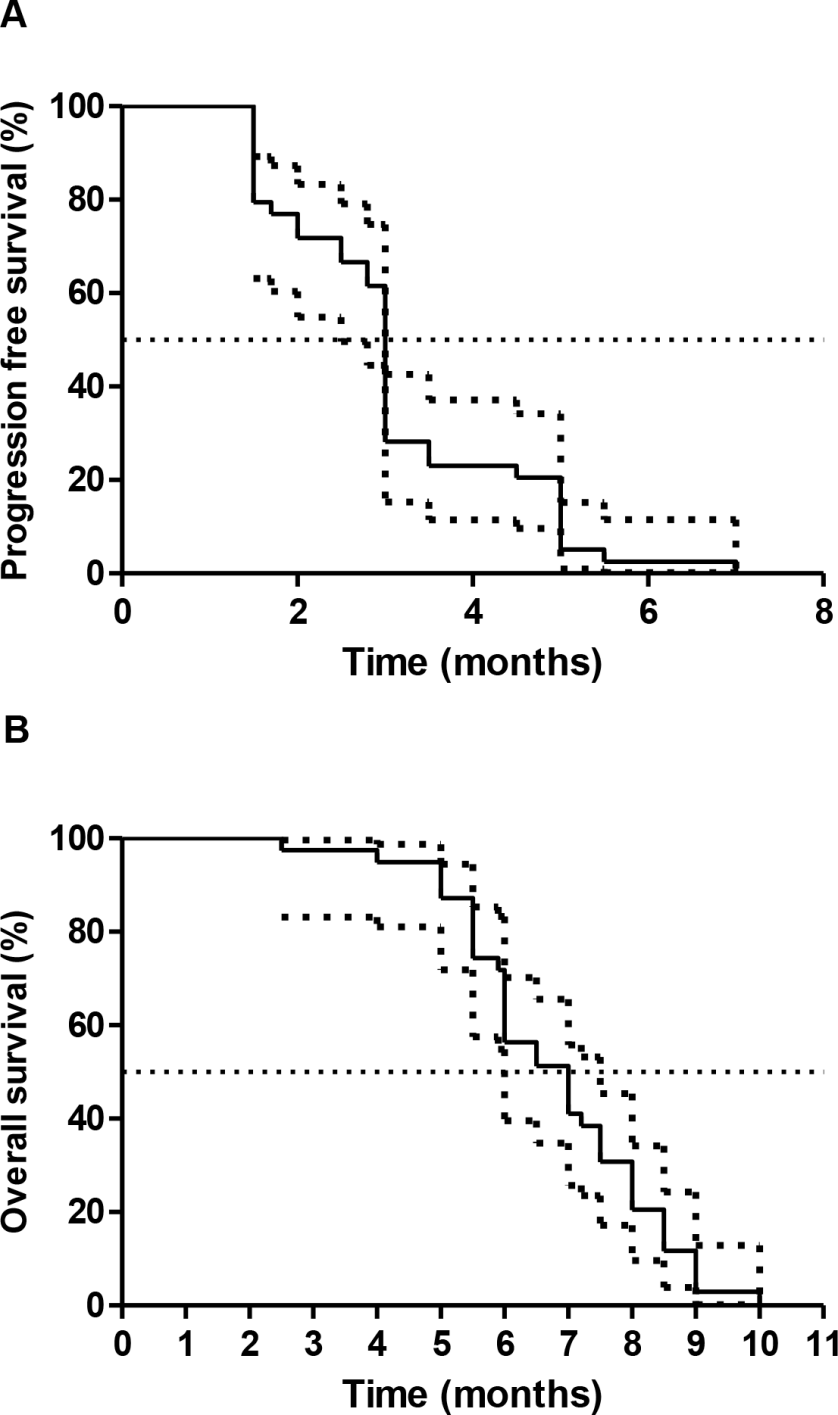
Kaplan-Meier survival Kaplan curves for progression-free survival A. and overall survival B. with 95% CI curves

At time for this analysis (January, 2015), all patients had progressive disease and 38 had died. Using the Kaplan-Meier method, median actuarial progression-free survival in all patients was 3.0 months (95% CI 2.92–3.09; Figure 1A). 3-month progression-free survival was 28.2% (95% CI 15.26–42.65); 6-month progression-free survival was 2.56% (95% CI 0.20–11.53). Median actuarial overall survival in all patients was 7 months (95% CI 5.99–8.03; Figure 1B). 6-month overall survival was 56.41% (95% CI 70.22–39.57).

### Adverse events

All 39 patients were assessable for toxicity (Table 3). 16 patients (41%) experienced one or more grade 3 or 4 toxicity during treatment (95% CI 25.6% to 57.9%). The most common single grade 3 or 4 toxicity was neutropenia (13 patients; 33%). Febrile neutropenia was experienced by 3 patients (7.7%). Other frequent grade 3 or 4 toxicities were nausea/vomiting (7 patients; 18%); neuropathy sensor (6 patients; 15.4%). There were no treatment-related deaths. In general, the toxicities were manageable through dose reduction, dose interruption, or supportive medical treatment.

**Table 3:** Toxicities of patients (*N* = 39)

Toxicity	Grade 1 or 2		Grade 3 or 4	
	No	%	No.	%
Anemia	8	20.5	3	7.7
Neutropenia	24	61.5	13	33
Thrombocytopenia	7	18	3	7.7
Febrile neutropenia	/	/	3	7.7
Central vein catheter related thrombosis	1	2.6	1	2.6
Nausea/vomiting	14	36	7	18
Diarrhea	8	20.5	2	5.2
Infection	6	15.4	4	10.3
Fever	2	5.2	1	2.6
Mucositis	5	13	2	5.2
Deep vein thrombosis	1	2.6	1	2.6
Neuropathy sensor	9	23	6	15.4

The effect of possible prognostic variables (baseline hemoglobin, ECOG performance status, albumin, presence of visceral metastasis) were examined in a univariate analysis. In the multivariable model, ECOG performance status of greater than 0, and presence of visceral metastasis were shown to significantly affect overall survival (Table 4). Hemoglobin and albumin level did not affect overall survival.

**Table 4:** Results of the multivariable Cox proportional hazard model for overall survival

	Hazard ratio (95% CI)	*p* value
ECOG performance status 1 versus 0	2.58 (1.21–6.31)	0.0026
Visceral metastases	7.21 (2.33–24.24)	0.001
Hemoglobin, less than 100 g/L versus at least 100 g/L	0.63 (0.29–1.62)	0.29
Albumin	1.65 (0.53–4.84)	0.414

Abbreviation: ECOG, Eastern Cooperative Oncology Group.

## DISCUSSION

Patients with distantly metastatic penile cancer are administered with chemotherapy as palliative therapy to control symptoms and prolong survival. Given that the management of metastatic penile cancer remains a formidable challenge, a clear need remains for systemic therapies for these patients, especially for those with good PS. This is the first prospective study that we know of with sufficient size to reliably estimate the outcomes of palliative chemotherapy for M1 penile carcinoma.

The TPF regimen had 38.5% objective response rate in 39 distantly metastatic penile cancer patients. Our results seem to be consistent with those of several other phase II studies in the advanced penile cancer setting [12, 19, 20]. A phase II study using similar regimen showed similar objective response rate. The major limitation in this study, as discussed by the authors, was that both patients with metastatic and locally advanced disease were included [20]. This facilitated a trial with efficient accrual but precluded examination with any subtlety of the differing requirements for palliative and neoadjuvant treatment. The clear requirement for the latter is a sufficiently high-response rate to allow for downstage; to reduce tumor size for improving the outcomes of resection and to eliminate microscopic distant metastases. While the purpose of palliative chemotherapy is to relieve symptoms and prolong survival. In this study using similar regimen to our trial, the authors reported considerable and troublesome toxicity: more than 20% patients experienced grade 3 or higher febrile neutropenia and diarrhea was prevalent in the patients. However, in our trial, these toxicities were moderate and manageable. Grade 3 or higher febrile neutropenia and diarrhea were only 7.7% and 5.2%, respectively. One reason for the difference might be the requirement of prophylactic growth factor support in our study. In addition, in the study by Nicholsons et al [20], patients with ECOG performance status 2 were also recruited. However, in our study, only ECOG performance status 0 to 1 were allowed. So the different general conditions of patients may also partially account for different toxicity profile. More importantly, we used lower dose of fluorouracil (500 mg/m^2/^d) compared with that in Nicholson's study. When fluorouracil 750 mg/m^2^/d in TPF regimen was used previously in other cancer treatments, gastrointestinal toxicities were the most common adverse events leading to dose reduction [15, 16]. And fluorouracil dose was most commonly reduced [16]. Because M1 stage penile cancer patients were more fragile compared with the locally advanced head and neck cancer patients for whom TPF was frequently given (the OS for the latter can be over 2 years.), lower dose of fluorouracil at 500 mg/m^2^ was given in our trial. This might be also a reason for the better tolerability in our trial.

Previously, there were several other phase II studies for advanced penile cancer treatments [3, 21]. The southwestern oncology group study of single-agent cisplatin was the largest phase II chemotherapy trial at that time, with 26 patients evaluable for response. All but 1 patient had stage IV disease, and none had undergone previous chemotherapy. An overall response rate of 15% was achieved. And the median overall survival time was 4.7 months [22].

In the largest published prospective study on advanced penile carcinoma, Haas et al. administered the BMP regimen in 40 evaluable patients with locally advanced or metastatic disease, with a response rate of 32%. However, grade 4 or 5 toxicity occurred in 28% of patients, which precluded general adoption of this regimen [23]. Retrospective data by Hakenberg et al confirmed the unacceptable toxicity profile of such a regimen [24].

The irinotecan/cisplatin study conducted by the European organization for research and treatment of cancer was prospective with 26 evaluable patients. However, the results were interpreted as negative by the investigators because the response rate had an 80% confidence interval that extended well below 30% [19].

The recent study by Pagliaro LC et al evaluated TIP as neoadjuvant chemotherapy for locally advanced penile cancer. An ORR of 50% was achieved and this regimen was also recommended for M1 stage penile cancer treatment [14]. However, generalizing such a neoadjuvant regimen to palliative setting would be unreliable. Thus, currently there is no standard chemotherapy regimen in the treatment of M1 penile cancer. The moderate ORR of TPF regimen showed here, together with the manageable toxicities and no treatment-related death, suggests this regimen could be a choice for these patients. The median OS of 7 months demonstrated the efficacy of this regimen is far from perfection and more effective combinations should be explored.

Understanding prognostic factors in M1 stage penile cancer receiving systemic chemotherapy may better define the population under study, facilitate future design of clinical trials, and help individualize patient treatment. Both the poor performance status and visceral metastases were shown to be independent negative prognostic factors in some malignancies [25]. A recent retrospective analysis of individual patient data of advanced penile cancer receiving first-line chemotherapy identified that visceral metastases and poor performance status were poor prognostic factors [26]. Our results were consistent with these results. Hemoglobin and albumin were not significant in multivariable analyses. These factors may be more relevant in localized small tumors undergoing curative surgery and not in patients with distant metastases.

The clinical efficacy noted might have been subject to the common biases noted in the phase II studies, such as selection bias, especially in the context of its small sample size. Thus the results should be interpreted with caution. Challenges facing studies in penile cancer include the time needed to accrue sufficient numbers of patients for a rare disease. Another recent trial of similar size in this disease took over 8 years to recruit 30 patients for neoadjuvant therapy [14]. In our trial, it took us less than 4 years for accruing 39 patients although M1 patients only comprise a minority in the overall patient population of penile cancer. As the largest cancer center in China, some of enrolled patients were referred to us from various local hospitals. The successful conduct of this trial showed that for this rare disease, accrual would not be an impossible barrier for clinical trials of penile cancer in developing countries. More input of health care resources from government and industry was necessary to facilitate this process and initiate further clinical studies.

In summary, the results obtained with this regimen are promising in terms of both efficacy and toxicity, especially in view of the very limited therapeutic options available for these patients. This regimen could be a choice for such patients and should be further confirmed in randomized clinical trials. In order to further prolong survival for this patient population, better understanding of tumor biology and study of novel combinations of biologic agents were warranted.

## PATIENTS AND METHODS

The study reported herein was a single-institution, open-label phase II trial that accrued patients in the Department of Internal Medicine in Shanghai Cancer Hospital, Fudan University, China. The clinical trial was registered in UMIN-CTR (UMIN000002697). The study was approved by the institutional review board, and patients were required to provide signed informed consent.

### Eligibility

Patients aged 18 to 75 years with histologically proven squamous carcinoma of the penis were required to have measurable disease as defined by the Response Evaluation Criteria in Solid Tumors (RECIST 1.1) [18]. Patients were required to be at stage M1 (distant metastases) according to 2002 TNM staging system of the American Joint Committee on Cancer with any T or any N stage.

Other main eligibility criteria included glomerular filtration rate (GFR) of 60 ml/min or greater, Eastern Cooperative Oncology Group (ECOG) performance status 0 to 1; life expectancy of 3 months or longer; absence of brain metastases; and adequate hepatic, and hematologic functions: absolute neutrophil count of 1.5 × 10^9^/L or more; platelet count greater than 100 × 10^9^/L; hemoglobin greater than 90 g/L; total bilirubin up to one and a half times the upper limit of normal; serum aspartate aminotransferase and alanine aminotransferase up to three times the upper limit of normal, or up to five times the upper limit of normal when liver metastases present.

Exclusion criteria included non-squamous cancer of the penis, primary squamous carcinoma of the urethra, previous chemotherapy in metastatic or adjuvant/neoadjuvant setting, and prior malignancy (other than squamous cell carcinoma or basal cell carcinoma of non-penile skin) in the previous 5 years. Patients were also excluded if they had uncontrolled infection; class II or higher congestive heart failure; pre-existing peripheral neuropathy of grade 1 or higher according to National Cancer Institute Common Terminology Criteria for Adverse Events (NCI-CTCAE) version 3 or ototoxicity of grade 1 or higher.

### Procedures

Chemotherapy consisted of docetaxel 75 mg/m^2^ (1-hour intravenous infusion) plus cisplatin 70 mg/m^2^ (1- to 3-hour intravenous infusion) on day 1, followed by fluorouracil 500 mg/m^2^/d (continuous intravenous infusion) for 5 days (TPF) every 3 weeks. The use of prophylactic granulocyte colony-stimulating factor was required at each cycle of chemotherapy. Dose modification criteria were pre-defined according to the previous report of TPF regimen in metastatic gastric cancer treatment [16]. All patients required steroid premedication before docetaxel, and antiemetic therapy in accordance with existing institutional policies for highly-emetogenic, docetaxel-based regimens. Treatment was administered until progressive disease, unacceptable toxic effects, death, or withdrawal from the study.

Before treatment, a complete medical history and physical examination were undertaken. Laboratory tests and tumor assessments were also performed. Tumor measurements were undertaken every 2 cycles on chemotherapy treatment. After completion of study treatments, clinical and radiological assessments were scheduled for every 2 months. Response of disease was assessed and confirmed by two investigators using RECIST, and it was required to be confirmed by follow-up radiological assessment at 4 weeks after initial assessment.

Adverse effects were graded and recorded according to NCI-CTCAE, version 3.0.

### Statistical analyses

The primary endpoint of this trial was the objective response to treatment (complete response plus partial response as defined by RECIST). Secondary endpoints included: toxicity; progression free survival and overall survival. PFS time was defined from the time of treatment initiation until clinically evident disease progression or death from any cause. OS was defined from the time of initiation of treatment to the date of death as a result of any cause.

The Simon minimax two-stage design was used to evaluate a null hypothesis that the true objective reponse rate (ORR) was ≤30% and an alternative hypothesis that the ORR was ≥50%, with a type 1 error (α) of 0.10 and type II error (β) of 0.10. The target accrual was 28 patients in the first stage, with an additional 11 patients to be enrolled in the second stage if ≥7 responses were observed. If ≥15 responses of 39, it would warrant further study. Survival curves were estimated using the Kaplan-Meier method, and 95% CIs for the medians were provided for progression-free survival and overall survival. A total of accrual of 43 patients was planned, with allowance for 10% dropout. Because we did not have any patient dropout, the 39 patients enrolled provided us with the desired evaluable patient sample size.

We used a univariate Cox proportional hazard model to search for significant prognostic factors of overall survival. Prognostic factors were baseline hemoglobin (≥100 g/L vs <100 g/L), ECOG performance (≥1 vs 0), albumin and presence of visceral metastasis. We used a backward stepwise selection procedure in the multivariable Cox proportional hazard analysis to find the most significant prognostic factors. We calculated hazard ratios (HRs) and 95% CIs for each significant prognostic factors. SAS version 9.2 was used for statistical analyses.
